# Effects of whey protein hydrolysate ingestion on post-exercise muscle protein synthesis compared with intact whey protein in rats

**DOI:** 10.1186/s12986-019-0417-9

**Published:** 2019-12-27

**Authors:** Kyosuke Nakayama, Ryoichi Tagawa, Yuri Saito, Chiaki Sanbongi

**Affiliations:** Food Microbiology and Function Research Laboratories, Meiji Co., Ltd., 1-29-1 Nanakuni, Hachiouji, Tokyo 192-0919 Japan

**Keywords:** Muscle protein anabolism, FSR, mTOR signaling, Leucine, Aminoacidemia

## Abstract

**Background:**

It is well known that ingestion of protein sources can stimulate muscle protein synthesis (MPS). The intake of whey protein is highly effective especially for accelerating MPS. Whey protein hydrolysate (WPH) can raise postprandial plasma concentration of amino acids, which impact stimulation of MPS more rapidly and highly than intact whey protein. However, it is unclear which is more effective for stimulating MPS, WPH or intact whey protein. The aim of the present study was to compare the effects of the WPH and whey protein on MPS in rats after exercise.

**Methods:**

Rats were first subjected to a 2 h. swimming protocol. After this, in experiment 1, we evaluated time-dependent changes in the fractional synthetic rate (FSR) of the triceps muscle in Male Sprague-Dawley rats after ingestion of intact whey protein (30, 60, 90 or 120 min after ingestion). Then in experiment 2, at the time point that the results of Experiment 1 revealed postprandial FSR was highest (60 min after ingestion), we measured the FSR after ingestion of the WPH or whey protein at two different doses (0.5 or 2.0 g protein/kg body weight), or with deionized water (control), again after exercise. Plasma components and mammalian target of rapamycin (mTOR) signaling were also measured.

**Results:**

In experiment 1, postprandial FSR was highest 60 min after whey protein was administered. In experiment 2, the FSR 60 min after ingestion of the WPH was higher than that of whey protein (significant treatment main effect). Moreover, at a lower dose, only the WPH ingestion caused greater MPS and phosphorylated 4E-binding protein 1 (4E-BP1) levels compared with the control group.

**Conclusion:**

These results indicate that ingestion of the WPH was associated with greater post-exercise MPS compared with intact whey protein, especially at lower doses.

## Background

Muscle protein turnover is a continuous cellular process. Muscle mass is regulated by changes in muscle protein synthesis (MPS) and to a lesser degree by muscle protein breakdown [1–3]. Intake of dietary protein, and in particular essential amino acids (EAA), produces a strong anabolic stimulus that elevates MPS through activation of the mammalian target of rapamycin (mTOR) complex 1 pathway [4–7]. Of the EAAs, leucine appears to be the amino acid responsible for activating MPS [8, 9]. Exercise is also well known to stimulate the rate of MPS [3, 10], and exercise and amino acid intake can have an additive effect on MPS [11, 12].

Whey protein is one protein source often used by athletes. The intake of whey protein is highly effective for accelerating muscle protein synthesis compared with other protein sources, such as casein [13–15], soy [14, 16] and wheat [16, 17]. This is probably because a rapid absorption rate [13, 18] and the leucine-rich amino-acid composition of whey protein lead to a rapid leucinaemia and aminoacidaemia that contribute to the acceleration of MPS.

Whey protein hydrolysate (WPH), which is produced by protease-mediated hydrolysis of intact whey protein, has an amino-acid composition identical to intact whey protein and can raise plasma concentration of amino acids more rapidly and to a higher level than intact whey protein after ingestion [19]. We have previously shown that WPH causes a greater increase in MPS than does a mixture of amino acids that is identical in amino acid composition [20]. Katsano et al. [21] also suggested that stimulation of the muscle protein anabolic response in the elderly was greater following ingestion of intact whey protein than ingestion of its constituent essential amino acid content, although it is well known that essential amino acids are primarily responsible for the amino acid-induced stimulation of muscle protein anabolism [6]. These results indicated it is possible that not only the amino-acid composition but also the difference of molecular form, such as amino acids, peptides or protein, affect skeletal muscle metabolism. Recently, Moro [22] showed that the ingestion of WPH induced a greater transport and accumulation of leucine into muscle, which increased amino acid utilization for protein synthesis compared with the ingestion of intact whey protein. However, it is unclear which is more effective for stimulating MPS, WPH or intact whey protein.

In the present study, we examined whether ingestion of WPH caused a greater increase in MPS after exercise compared with intact whey protein. First, we investigated the measurement conditions for comparison of WPH and whey protein to evaluate time-dependent changes of muscle protein synthesis rate after ingestion of whey protein. Next, we compared the effects of WPH versus intact whey proteins on MPS at two different doses.

## Materials and methods

### Animals

Male Sprague-Dawley rats with body weights (BW) of approximately 150 g (CLEA Japan, Inc., Tokyo, Japan) were used in this study. The animals were maintained at 22 ± 2 °C, with lights on from 07.00 to 19.00 h and off from 19.00 to 7.00 h, and had free access to food (protein 23.6%, fat 5.3%, carbohydrate 54.4%, ash 6.1%, fibre 2.9% and moisture 7.7%; MF; Oriental Yeast Company Ltd., Tokyo, Japan) and water. The study was approved by the Animal Committee of the Food Science Research Laboratory, Meiji Company Limited, with animals receiving care according to the guidelines laid down by this committee (protocol nos. 2014_3871_0204, 0205, 0206, 0268, and 0269).

### Experiment 1: evaluation of time-dependent effects of intact whey protein on MPS

The swimming exercise protocol matched a protocol previously described [23]. Two days before the experiment, rats were acclimated to swimming exercise for 30 min. On the day of the experiment, food-deprived (16 h) rats swam for 2 h, with four rats swimming simultaneously in a barrel filled to a depth of 50 cm, allowing an average surface area of 400 cm^2^ for each rat. Water temperature was maintained at a constant 34 °C during the swimming protocol. Immediately following exercise, rats were given oral administration of whey protein concentrate (WPC) (Tatua Co-operative Dairy Co., Ltd., Morrinsvill, New Zealand) solution (2.0 mL/100 g BW, amounts of protein were 2.0 g/kg BW). Rats (*n* = 8~9/time point) were then euthanized 30, 60, 90 and 120 min after administration. One group of rats was euthanized 15 min after exercise without administration of test solution (exercise controls, *n* = 7). Rats were anesthetized with 30% isoflurane diluted with propylene glycol and euthanized by exsanguination. The triceps muscles were excised and stored at − 80 °C until further use.

### Experiment 2: comparison of WPH and intact whey protein

As mentioned above, the rats swam for two hours on the day of the experiment. Immediately following exercise, WPH (Meiji Co., Ltd., Tokyo, Japan), WPC (2.0 mL/100 g BW, amounts of protein were 0.5 or 2.0 g/kg BW, *n* = 8/treatment/dose) or deionized water (controls, *n* = 8) was administered to the rats as a single dose. Individual rats were anesthetized and euthanized 60 min after ingestion. This was the time point at which muscle protein synthesis rate following ingestion of WPC was highest in Experiment 1. Blood was taken from the inferior vena cava and plasma collected. The triceps muscles were excised and stored at − 80 °C until further use.

The WPH used in the present study had an average peptide length of 3.64, which was calculated as the ratio of the total nitrogen/amino nitrogen in the protein samples. The composition of WPH and WPC are presented in Table 1.

**Table 1:** The composition of WPH and WPC

	WPH	WPC
	g/100 g sample	
Carbohydrate	6.5	5.1
Fat	0.1	5.7
Protein	84.1	80.2
	g/100 g protein	
Alanine	4.58	4.55
Arginine	3.17	3.24
Aspartic acid + Asparagine	11.34	10.57
Cystine	2.40	2.64
Glutamic acid + Glutamine	15.36	15.60
Glycine	1.99	1.77
Histidine	2.44	2.32
Isoleucine	5.34	5.71
Leucine	11.85	12.78
Lysine	9.40	9.06
Methionine	2.13	2.15
Phenylalanine	3.32	3.60
Proline	4.61	4.42
Serine	5.04	4.72
Threonine	5.28	5.04
Tryptophan	3.03	2.98
Tyrosine	3.83	3.79
Valine	4.88	5.06

### Administration of metabolic tracer

Fifteen min before killing, a bolus dose (45 mg/kg body weight, 22.5 mg/ml) of ^2^H-labelled phenylalanine ([^2^H_5_]Phe; Cambridge Isotope Laboratories, Inc., Tewksbury, MA, USA) was injected via the tail vein for measurement of fractional protein synthesis rate (FSR). Fifteen min after injection, the rats were euthanized, and the triceps muscles were excised and frozen rapidly. The elapsed time from injection until freezing was recorded as the actual time for incorporation of the labelled amino acid into the protein.

### Measurement of muscle protein synthesis (MPS)

The rate of MPS was determined by measuring the incorporation of the injected [^2^H_5_] Phe into the triceps muscle proteins, using a procedure described previously [20]. Briefly, frozen triceps muscle samples (560–798 mg) were weighed and homogenized in ice-cold 3% ((w/v)) perchloric acid. After centrifugation, the supernatants were collected and the pellets were further washed with distilled water and hydrolyzed with hydrochloric acid. The enrichment of [^2^H_5_] Phe in intramuscular free amino acids was measured in the supernatants and the muscle protein-bound [^2^H_5_] Phe enrichment was measured in the hydrolyzed muscle protein pellets using liquid chromatography tandem-mass spectrometry (ACQUITY TQD, Waters Corporation, Milford, MA, USA). We calculated the FSR, defined as the percentage of muscle protein renewed each day, of triceps muscle proteins according to the formula:

$$ \mathrm{FSR}=\left(\mathrm{Eb}\times 100\right)/\left(\mathrm{Ea}\times \mathrm{t}\right) $$

where Eb is the protein-bound Phe enrichment, Ea is the Phe enrichment in the free intramuscular pool, and t is the time interval between the injection of the tracer and the cooling of the muscle sample, expressed in days.

### Plasma measurements

In Experiment 2, plasma insulin levels were measured using a commercial ELISA kit for rat insulin (Mercodia Rat Insulin ELISA, Mercodia AB, Uppsala, Sweden). Plasma amino acids were measured by high-performance liquid chromatography with pre-column 6-aminoquinolyl-N-hydroxysuccinimidyl carbamate derivatization [19].

### Western blot analysis

Western blot analysis was performed using a procedure described previously [23]. In Experiment 2, triceps muscle samples were used to measure phosphorylation status of mTOR (Ser2448), ribosomal protein S6 kinase (S6K1 Thr389), and 4E-binding protein 1 (4E-BP1 Thr37/46) (Cell Signaling Technology, Danvers, MA, USA). Phosphorylation levels were determined by expression of phosphorylated protein divided by expression of nonphosphorylated total protein. Expression levels of β-actin (Cell Signaling Technology, Danvers, MA, USA) were also measured.

### Statistical analysis

All values are expressed as mean ± standard error of the mean (SEM). In Experiment 1, the data were analyzed using a one-factor (1 × 5) analysis of variance (ANOVA) with a Tukey’s post hoc test. In experiment 2, the data were first analyzed using Bonferroni corrected *t* tests for comparison of the treatment (WPH or WPC) group with the control group. The data were then analyzed using a two-factor (2 × 2; treatment, dose) ANOVA. Differences among individual means were assessed using Tukey’s post hoc test when significant interactions between treatment and dose were found. The expression levels of β-actin were analyzed using a one-factor (1 × 5) ANOVA. Bonferroni corrections were calculated using Microsoft Excel (Microsoft Corp., Redmond, WA, USA) and the other analyses were performed using SPSS for Windows, version 23 (IBM Japan, Ltd., Tokyo, Japan). Significance was set at *p* <  0.05.

## Results

### Experiment 1

#### Fractional synthetic rate (FSR)

**Fig. 1:**
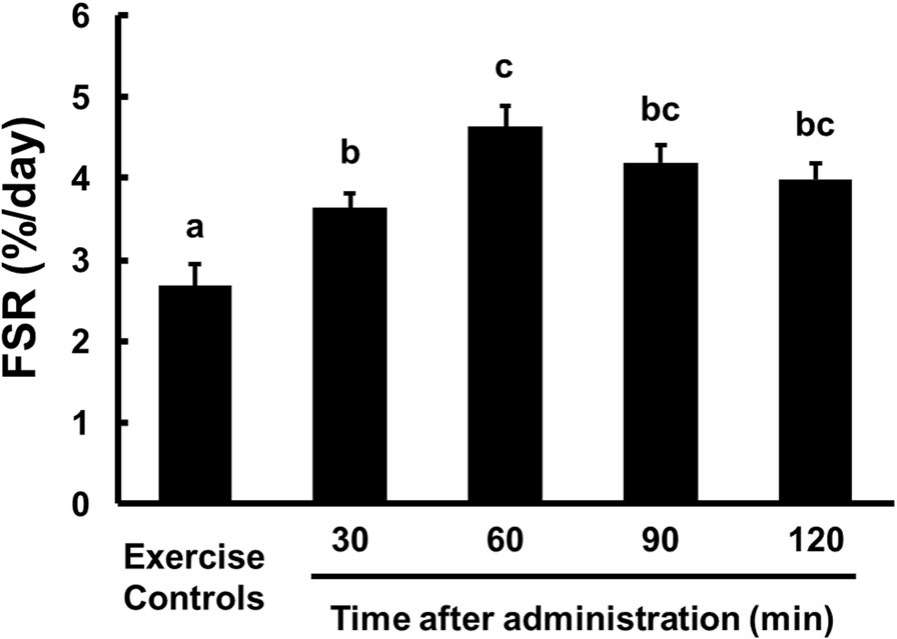
The FSR of triceps muscle tissue from rats administered WPC. Data are presented as means ± SEM (n = 7–9). ^a, b, c^Mean values with unlike letters were significantly different, *p* < 0.05; Tukey’s post hoc test

Triceps muscle protein FSR data are presented in Fig. 1. Compared with the exercise control group, the FSR was significantly higher (*p* <  0.05) in the WPC group at each time point. In the WPC group, the FSR 60 min after administration was significantly higher than the FSR 30 min after administration. Postprandial FSR was highest 60 min after WPC was administered (3.64 ± 0.19, 4.62 ± 0.28, 4.19 ± 0.21 and 3.99 ± 0.19%/day for 30, 60, 90 and 120 min).

### Experiment 2

#### Fractional synthetic rate (FSR)

**Fig. 2:**
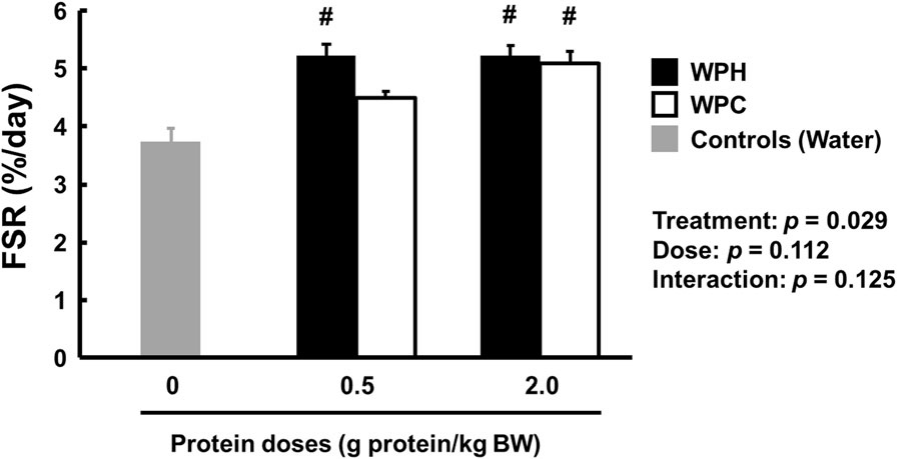
The FSR of triceps muscle tissue from rats administered WPH, WPC or water, measured 60 min after administration. Data are presented as means ± SEM (n = 8). ^#^ Significantly different from the control group, *p* < 0.05; Bonferroni corrected *t* tests

Triceps muscle protein FSR data are presented in Fig. 2. Compared with the control group, the FSR was significantly higher (*p* <  0.05) in the WPH groups at both doses and in the WPC group at a dose of 2.0 g/kg BW. The FSR in the WPH groups was significantly higher than that of the WPC groups (main effect for treatment: *p* <  0.05; treatment × time: *p* = 0.125).

### Plasma amino acid and insulin levels

Plasma EAA, leucine and insulin levels are presented in Table 2. The plasma EAA and leucine levels in the WPC groups at both doses and in the WPH group at a dose of 2.0 g/kg BW were significantly higher than in the control group (*p* < 0.05). There were no significant differences between the treatment (WPH or WPC) groups and control group in the plasma insulin levels. Significant treatment × interactions (*p* < 0.05) were found in the plasma leucine and insulin levels. The WPC group had significantly higher (*p* < 0.05) plasma leucine levels compared with the WPH group at a dose of 2.0 g/kg BW.

**Table 2:** Plasma EAA, leucine and insulin levels in rats, measured 60 min after administration^1^

	Group·Protein doses (g protein/kg BW)					*p* (two-factor ANOVA)		
	Controls·0	WPH·0.5	WPC·0.5	WPH·2.0	WPC·2.0	Treatment	Dose	Interaction
EAA (μmol/L)	1907 ± 39	2034 ± 35	2221 ± 59^#^	4994 ± 179^#^	5322 ± 287^#^	0.147	< 0.001	0.684
Leu (μmol/L)	226 ± 11	252 ± 7^a^	309 ± 15^a,#^	850 ± 32^b,#^	1047 ± 57^c,#^	0.001	< 0.001	0.049
Insulin (pmol/L)	5.3 ± 0.5	6.5 ± 0.4^ab^	4.4 ± 0.8^a^	7.5 ± 1.3^ab^	10.5 ± 1.7^b^	0.668	0.004	0.036

^1^Values are mean ± SEM. ^a, b, c^Mean values with unlike letters were significantly different, *p* < 0.05; Tukey’s post hoc test. ^#^ Significantly different from Controls·0, *p* < 0.05; Bonferroni corrected *t* tests

### Phosphorylated mTOR, S6K1 and 4E-BP1 levels

**Fig. 3:**
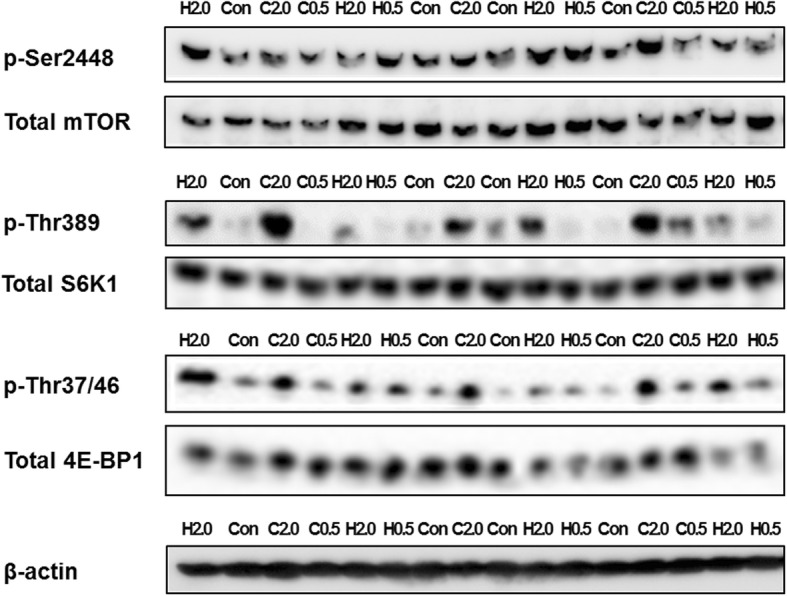
Representative western blotting images of mTOR Ser2448, S6K1 Thr389, 4E-BP1 Thr37/46 and β-actin in rats administered WPH, WPC or water, measured 60 min after administration. Con, control group; H0.5, WPH at a dose of 0.5 g/kg BW; C0.5, WPC at a dose of 0.5 g/kg BW; H2.0, WPH at a dose of 2.0 g/kg BW; C2.0, WPC at a dose of 2.0 g/kg BW

Phosphorylated mTOR (Ser2448), S6K1 (Thr389) and 4E-BP1 (Thr37/46) levels are presented in Table 3, and the representative western blotting images are presented in Fig. 3. The expression levels of β-actin were similar among all groups. At a dose of 2.0 g/kg BW, the phosphorylated 4E-BP1 levels in both groups and the phosphorylated S6K1 levels only in the WPC group were significantly higher than in the control group (*p* < 0.05). At a dose of 0.5 g/kg BW, the phosphorylated 4E-BP1 levels only in the WPH group were significantly higher than in the control group (*p* < 0.05). No significant interactions were found in the phosphorylated mTOR, S6K1 and 4E-BP1 levels.

**Table 3:** Western blotting analyses of synthesis-associated signaling proteins in rats, measured 60 min after administration^1^

	Group·Protein doses (g protein/kg BW)				*p* (two-factor ANOVA)		
	WPH·0.5	WPC·0.5	WPH·2.0	WPC·2.0	Treatment	Dose	Interaction
*Phosphorylated/total, fold of control group*							
mTOR Ser2448	1.04 ± 0.11	1.06 ± 0.09	1.45 ± 0.31	1.80 ± 0.28	0.425	0.015	0.472
S6K1 Thr389	0.98 ± 0.08	1.24 ± 0.09	1.78 ± 0.33	2.59 ± 0.44^#^	0.068	0.001	0.350
4E-BP1 Thr37/46	2.03 ± 0.23^#^	1.51 ± 0.16	3.25 ± 0.39^#^	4.55 ± 0.83^#^	0.424	< 0.001	0.069

^1^Values are mean ± SEM. # Significantly different from Controls·0, *p* < 0.05; Bonferroni corrected *t* tests

## Discussion

The main purpose of the present study was to investigate the effects of ingestion of WPH on MPS compared with that of intact whey protein at two different doses. In the present study, we first evaluated time-dependent changes of MPS after ingestion of intact whey protein, and we found that the FSR reached a peak at 60 min after whey protein ingestion in rats. Then, we compared the FSR at 60 min after ingestion of the WPH with that of the WPC at two different doses. As a result, the FSR in the WPH groups was higher than in the WPC groups (significant treatment main effect). Moreover, at a lower dose (0.5 g protein/kg BW), only the WPH ingestion caused greater MPS compared with water ingestion (As supplemental data, when we analyzed the FSR data at a lower dose using *t* tests, the *p*-value between WPH group and WPC group was 0.011). The primary finding from our study is that the WPH ingestion was associated with greater post-exercise MPS than intact whey protein ingestion, especially at lower doses. It already was known that ingestion of whey protein produces greater muscle anabolic response compared with other protein sources [13–17], but large amounts of whey protein is required to be ingested to maximize postprandial MPS [24–26]. WPH may be beneficial for people who have a difficult time consuming sufficient amounts of protein to maintain or increase muscle mass, such as older adults, certain patients, and athletes because the WPH may maximize MPS at lower doses than intact whey protein and other protein sources.

Elevation of the concentrations of plasma EAA, especially that of leucine, may be one of the key factors for stimulating MPS [13, 27]. Additionally, a rapid acute rise in postprandial circulating EAA or leucine levels following consumption of protein-rich food has been associated with an increase in MPS [23, 28, 29]. Previous studies showed that the ingestion of WPH was associated with a greater increase of plasma amino acids compared with intact whey protein [19] or a free amino acid mixture [30]. In the present study, the plasma EAA and leucine levels 60 min after ingestion of intact whey protein at a lower dose were significantly higher than those of the control group, although they were not increased with WPH. At a higher dose (2.0 g protein/kg BW), the plasma leucine levels in the WPC group were significantly higher than in the WPH group. As a whole, the plasma amino acid levels in the WPC groups appeared to be higher than in the WPH group 60 min after administration. At first glance, these look like unexpected results, but these results are really consistent with the previous study [19]. We speculate that the plasma amino acid levels in the WPH groups reached their peaks earlier (e.g. 20 or 30 min after administration) and became higher (and also declined earlier) than in the WPC groups as is the case with the previous study [19]. As mention above, elevation of plasma leucine levels is associated with MPS stimulation: the ‘leucine trigger’ hypothesis states that there may be a threshold level of leucine to trigger MPS [31]. It’s possible that only the WPH group exceeded the threshold level of leucine to trigger MPS at a lower dose. That may be one of the reasons why the WPH ingestion was associated with greater stimulation of MPS than intact whey protein ingestion especially at lower doses. However, we have not measured the plasma amino acid levels earlier than 60 min after administration. Moro [22] indicated that the ingestion of WPH induced similar levels of aminoacidemia compared with that of intact whey protein, which is inconsistent with our hypothesis, so further study is necessary to confirm it.

Insulin is also a potent anabolic stimulus to promote MPS [32], which increases following protein ingestion [33]. In the present study, plasma insulin levels did not differ between the control group and the WPH or the WPC groups 60 min after administration. That’s probably because of rapid increases and decreases of plasma insulin levels (within 60 min after administration) in response to protein ingestion as seen in the previous studies [22, 23, 30].

We analyzed the phosphorylation status of mTOR signaling proteins, which are important modulators of MPS [4], to support the results of FSR. Eukaryotic initiation factor 4E-BP1 is one of the downstream targets of mTOR, and in the present study, phosphorylation of 4E-BP1 in the WPH group at a lower dose was significantly higher than in the control group, although the WPC group at a lower dose was not. Increasing phosphorylation of 4E-BP1 in the WPH group would be related to enhanced MPS at a lower dose. Unlike the response of 4E-BP1, phosphorylation of S6K1, which is also one of the downstream targets of mTOR, increased only in the WPC group at a high dose compared with the control group. Phosphorylation of mTOR showed similar pattern to that of S6K1 (phosphorylation of mTOR in the WPC group at a high dose tended to be higher than control group, *p* = 0.063). Although both 4E-BP1 and S6K1 are downstream targets of mTOR, several previous studies have reported a discrepancy of phosphorylation between 4E-BP1 and S6K1, with phosphorylation of 4E-BP1 delayed relative to S6K1 and plasma amino acid response [23, 34, 35]. Although amino acids, particularly leucine, can activate mTOR signaling [36–38], 4E-BP1 may not be as responsive to leucine as mTOR and S6K1. Moura [39] showed that leucyl-valine and valyl-leucine, which are dipeptides contained in WPH [40], strongly increased phosphorylation of 4E-BP1. Therefore some active component contained in the WPH, such as bioactive peptides, may contribute to activating 4E-BP1 independently of mTOR, which is activated by leucine, and then stimulating MPS. It is also possible that we could not detect the peak of activation of mTOR and S6K1 as is the case with plasma EAA and leucine levels, and that may be associated with stimulating MPS.

In the present study, we compared the effects of the WPH ingestion on MPS with that of intact whey protein at the time point when the FSR reached a peak after ingestion of intact whey protein. Therefore, we consider the experimental design in the present study appropriate for evaluation of FSR. However, consideration of the mechanisms involved in the results of FSR will require evaluation at other time points, especially earlier than in the present study. That is the limitation of this study.

## Conclusion

In conclusion, these results demonstrate that ingestion of the WPH was associated with greater post-exercise MPS compared with intact whey protein, especially at lower doses. The increase of phosphorylation of 4E-BP1, which is one of the downstream targets of mTOR, may be related to the stimulation of MPS when WPH is ingested at lower doses. The difference of postprandial aminoacidemia or certain bioactive peptides contained in the WPH might be related to greater stimulation of MPS following WPH ingestion. Although intact whey protein is consumed worldwide and it is well known that intact whey protein intake is highly effective for stimulating MPS, WPH may be an alternative nutrition strategy to improve protein synthesis and impact muscle mass maintenance.

## Data Availability

The datasets during and/or analyzed during the current study available from the corresponding author on reasonable request.

## Abbreviations

4E-BP1: 4E-binding protein 1
ANOVA: Analysis of variance
BW: Body weight
EAA: Essential amino acids
FSR: Fractional synthetic rate
MPS: Muscle protein synthesis
mTOR: Mammalian target of rapamycin
S6K1: Ribosomal protein S6 kinase
SEM: Standard error of the mean
WPC: Whey protein concentrate
WPH: Whey protein hydrolysate
